# Root-Knot and Cyst Nematodes Activate Procambium-Associated Genes in *Arabidopsis* Roots

**DOI:** 10.3389/fpls.2017.01195

**Published:** 2017-07-13

**Authors:** Yasuka L. Yamaguchi, Reira Suzuki, Javier Cabrera, Satoru Nakagami, Tomomi Sagara, Chika Ejima, Ryosuke Sano, Yuichi Aoki, Rocio Olmo, Tetsuya Kurata, Takeshi Obayashi, Taku Demura, Takashi Ishida, Carolina Escobar, Shinichiro Sawa

**Affiliations:** ^1^Graduate School of Science and Technology, Kumamoto University Kumamoto, Japan; ^2^Facultad de Ciencias Ambientales y Bioquímica, Universidad de Castilla – La Mancha Toledo, Spain; ^3^Graduate School of Biological Science, Nara Institute of Science and Technology Ikoma, Japan; ^4^Graduate School of Information Sciences, Tohoku University Sendai, Japan; ^5^Plant Global Education Project, Graduate School of Biological Science, Nara Institute of Science and Technology Ikoma, Japan

**Keywords:** plant-parasitic nematodes, RNA-sequencing, procambial cells, root-knot nematode, cyst nematode, *M. incognita*

## Abstract

Developmental plasticity is one of the most striking features of plant morphogenesis, as plants are able to vary their shapes in response to environmental cues. Biotic or abiotic stimuli often promote organogenesis events in plants not observed under normal growth conditions. Root-knot nematodes (RKNs) are known to parasitize multiple species of rooting plants and to induce characteristic tissue expansion called galls or root-knots on the roots of their hosts by perturbing the plant cellular machinery. Galls contain giant cells (GCs) and neighboring cells, and the GCs are a source of nutrients for the parasitizing nematode. Highly active cell proliferation was observed in galls. However, the underlying mechanisms that regulate the symptoms triggered by the plant-nematode interaction have not yet been elucidated. In this study, we deciphered the molecular mechanism of gall formation with an *in vitro* infection assay system using RKN *Meloidogyne incognita*, and the model plant *Arabidopsis thaliana.* By taking advantages of this system, we performed next-generation sequencing-based transcriptome profiling, and found that the expression of procambium identity-associated genes were enriched during gall formation. Clustering analyses with artificial xylogenic systems, together with the results of expression analyses of the candidate genes, showed a significant correlation between the induction of gall cells and procambium-associated cells. Furthermore, the promoters of several procambial marker genes such as *ATHB8*, *TDR* and *WOX4* were activated not only in *M. incognita*-induced galls, but similarly in *M. javanica* induced-galls and *Heterodera schachtii*-induced syncytia. Our findings suggest that phytoparasitic nematodes modulate the host’s developmental regulation of the vascular stem cells during gall formation.

## Introduction

**FIGURE 1:**
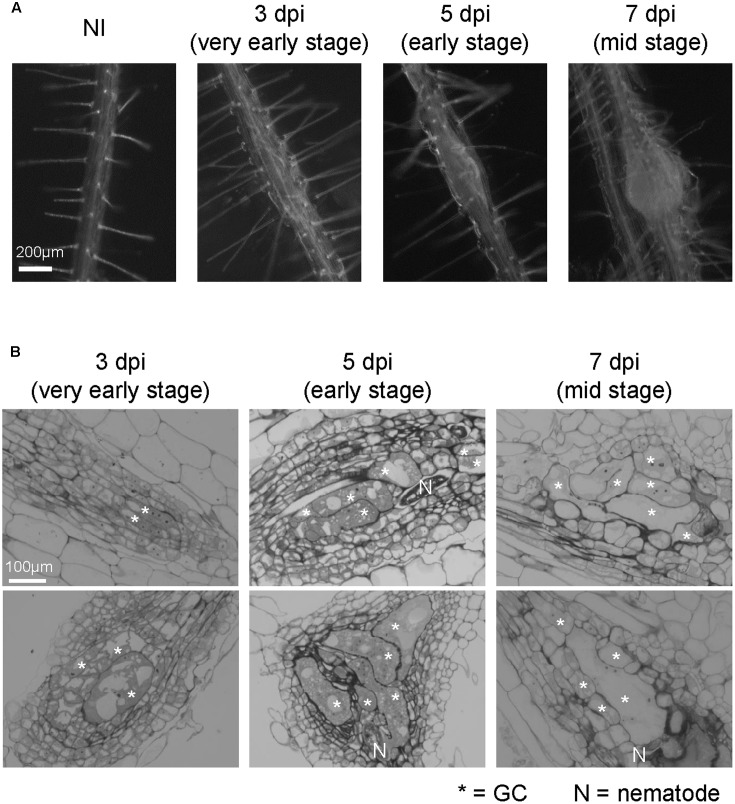
Growth dynamics of *M. incognita*-induced galls in the *in vitro* assay system. **(A)** Representative images of non-infected roots (NI) and galls at 3, 5, and 7 dpi. Roots were grown on an agar medium and then inoculated with surface-sterilized RKNs 5 days after germination. Scale bar = 200 μm. **(B)** Histological characterization of 3, 5, and 7 dpi galls. Upper panels show sections obtained from smaller galls, lower panels show sections obtained from larger galls. Scale bar = 100 μm. Asterisks indicates GCs, N indicates RKN.

Plant roots play important roles by supporting the plant in the soil and taking up nutrients for growth. Furthermore, the roots are the frontline of plant-microbe interactions with the various microorganisms that live in the soil. Plant-parasitic nematodes, such as the RKNs and the CNs infect and feed on the plant roots. Second-stage juvenile (J2) larvae of RKN invade the host plant roots and to reach the vasculature. The RKNs then inject various effector proteins into vasculature cells, which convert these cells into specialized feeding sites (GCs). In some host-species such as *Arabidopsis*, mature females become substantially larger such that they break through the root tissue and become exposed on the root surface. In a single feeding site, normally several GCs and many surrounding NCs are induced, leading to a bulging section on the root known as a gall or root-knot (Escobar et al., 2015; **Figure 1**). GCs are enlarged, metabolically active multinuclear cells that nourish RKNs to develop and reproduce (**Figure 1B**). Based on their morphological features, GCs are generated via atypical cell cycle events that is a process chromosome duplication and nuclear divisions without cytokinesis (Gheysen and Mitchum, 2009; de Almeida Engler et al., 2011). In addition to the GCs and NCs, surplus xylem and Ph are also produced in the feeding site during the later stages of gall formation (Bartlem et al., 2014). LATERAL ORGAN BOUNDARIES-DOMAIN 16 (LBD16), one of a key component for lateral root formation, is thought to be involved in RKN feeding site formation (Cabrera et al., 2014). However, the molecular mechanisms underlying the irregular organogenesis induced by RKN are not yet well-understood.

The plant vasculature plays an important role as it transport water, sugars, nutrients and signaling molecules, while the mature xylem also provides mechanical support to the plant through its thick secondary cell walls. The first step of vasculature development is the differentiation of procambial cells during early embryogenesis, whereas the Ph, xylem cell fates are specified by the end of embryogenesis. The procambium is present between the xylem and the Ph, and function as a source of cells that are fated to differentiate into xylem or Ph (Miyashima et al., 2013). Thus, the procambial cells are thought to be multipotent stem cells. This multipotency of the procambium is retained after the establishment of the vasculature, hence the vasculature maintains its own stem cell pool throughout the life of the plant (Miyashima et al., 2013).

Recent studies have shown that various phytohormones play crucial roles in generating the positional cues for vasculature formation in *Arabidopsis thaliana* embryogenesis (De Rybel et al., 2016). In particular, auxin is known to be a key regulator of vascular development. The expression of *AUXIN RESPONSE FACTOR 5* (*ARF5*) / *MONOPTEROS* (*MP*) is triggered during early embryogenesis (Hardtke and Berleth, 1998). The signaling modules that function downstream, e.g., TARGET OF MONOPTEROSs (TMOs) and class III homeodomain-leucine zipper (HD-Zip III) proteins, are employed to specify the appropriate developmental program for each cell type (Donner et al., 2009; Schlereth et al., 2010). Among the TMOs, *TMO6* (also known as *DOF5.3*) has been shown to be expressed in the stele (Gardiner et al., 2010). Although various types of DOF-class transcription factors are expressed in stele, their contribution to vascular development are currently unclear. In contrast, ATHB8 is a member of the HD-ZIP III class of transcription factors. ATHB8, together with other HD-ZIP III TFs such as REVOLUTA (REV), PHABULOSA (PHB), PHAVOLUTA (PHV) and CORONA (CNA)/ATHB15, are involved in the cell fate specification of xylem precursors in the root meristem (Carlsbecker et al., 2010; Miyashima et al., 2011).

After the vasculature has been established, the balance between cell proliferation and cell differentiation in the procambium is regulated by a ligand-receptor-mediated intercellular signaling system composed of TRACHEARY ELEMENT DIFFERENTIATION INHIBITORY FACTOR (TDIF) and its cognate receptor TDIF receptor (TDR) / Phloem INTERCALATED WITH XYLEM (PXY) (Hirakawa et al., 2008). As a member of the CLE family, TDIF is a small signaling peptide that is encoded by *CLE41* and *CLE44*. On the other hand, TDR is a leucine-rich repeat receptor-like protein kinase (LRR RLK). *CLE41* is expressed in the Ph, while *TDR* is expressed in the procambium. When TDIF is perceived through the extracellular domain of the TDR, the intracellular kinase domain activates the intracellular signaling pathway to induce the expression of the *WUSCHEL-RELATED HOMEOBOX* (*WOX*) family transcription factor *WOX4* (Hirakawa et al., 2010). This TDIF-TDR-WOX4 signaling pathway is required for the precise regulation of procambial cell proliferation, thus defects in this pathway are known to cause reduction of procambium.

Recently CN has been shown to modulate the procambial cell proliferation of feeding cell formation through the TDIF-TDR-WOX4 signaling pathway (Guo et al., 2017). However, no equivalent TDIF-class CLE peptides were identified in RKNs, further highlighting the differences in the regulatory mechanisms between CNs and RKNs during feeding site formation. On the other hand, similarities between early *Meloidogyne javanica*-induced GCs and cell suspensions differentiating into xylem were shown with transcriptomic analysis (Barcala et al., 2010). However, the importance of vasculature development during gall formation, including the generation of RKN-induced GCs and NCs remains unclear.

In this study, we validated that cell proliferation and initiation of gall formation occurred in the vasculature during RKN (*M. incognita*) infection through histological analyses. Furthermore, we conducted transcriptome profiling for different stages of gall formation induced by RKN (*M. incognita*), which showed that the global transcriptional profiles of galls more closely resemble those of the procambium, than those of xylem or the Ph. Our transcriptome analyses, validated by q-PCR and procambial marker promoter:GUS histochemical observations also demonstrated the activation of procambium-associated genes in galls induced by RKN (*M. incognita*). In addition, these transcripts were also detected in *M. javanica*-induced galls and *Heterodera schachtii*-induced syncytia. These results suggest a common mechanism regulate both gall formation and syncytium formation during RKN and CN infection. Based on our findings, we propose that nematodes first increase the procambial stem cell pool and then induce the feeding site.

## Materials and Methods

### Plant Materials

*Arabidopsis thaliana* transgenic lines with *AtHB8pro:GUS, TDRpro:GUS* or *WOX4pro:GUS* were previously described in Baima et al. (1995) and Hirakawa et al. (2010), respectively. The mutants used in this study were provided by Yuki Hirakawa [*tdr-1*/*pxy-5*, *wox4-1* (Hirakawa et al., 2010)] and Enrico Scarpella [*athb8-11* (Prigge et al., 2005)]. All lines were recorded in the Columbia (Col-0) accession.

### Nematode Infection Assays

*Meloidogyne incognita* juveniles were prepared as previously described (Nishiyama et al., 2015). *Arabidopsis* seeds were surface-sterilized and germinated on quarter-strength MS media (Murashige and Skoog Basal Medium, Sigma) containing 0.5% sucrose (Wako 196-00015) and 0.6% Phytagel (Sigma P8169) at pH 6.4, in 9 cm × 9 cm square Petri dishes under continuous light at 23°C. After inoculation of the 80 J2 larvae to 5 days after germination *Arabidopsis* seedlings, the roots were covered with black paper and incubated under the short-day condition (8 h light/ 16 h dark) at 25°C. *M. javanica* and *H. schachtii* juveniles were obtained from cucumber and mustard roots, respectively, as previously described (Bohlmann and Wieczorek, 2015; Díaz-Manzano et al., 2016); respectively. The *Arabidopsis* transgenic lines were inoculated with these nematode species, grown vertically for 5 days under the long-day condition (16 h light/8 h dark) at 23°C and inoculated with 10–15 juveniles per plant according to the methods described (Cabrera et al., 2014; Olmo et al., 2017).

### Histological Analyses

At 3, 5, and 7 dpi, the galls were fixed in a mixture of 2% glutaraldehyde (v/v) and 20mM cacodylic acid overnight in 4°C, dehydrated, embedded in Technovit 7100 (Heraeus Kulzer) and sectioned at 6 μm thickness with an RM2255 microtome (Leica Biosystems). Sections were briefly stained with Toluidine Blue, observed using an Axio Imager M1 microscope and photographed using a DP71 Digital camera.

### RNA-Sequencing (RNA-Seq)

Total RNA was extracted from the galls at 3, 5, and 7 dpi (**Figure 1A**), or from non-inoculated roots (NIs) with the RNeasy micro kit (QIAGEN, United States) according to the manufacturer’s protocol. The gall diameters were less than 190 μm at 3 dpi, 190–380 μm at 5 dpi and 380–540 μm at 7 dpi. Two independent biological replicates were analyzed for each time point. Three replicates for the RNA samples would have been ideal, however, it is difficult to prepare large quantities of gall tissue samples for RNA seq analyses due to their small size and the manually intensive nature of their dissection. The quality of the RNA samples was examined using an Agilent RNA 6000 Pico LabChip Kit in an Agilent 2100 Bioanalyzer (Agilent, Santa Clara, CA, United States). The mRNA fractions were isolated from 500 ng total RNA with the NEBNext Poly(A) mRNA Magnetic Isolation Module (New England BioLabs, Ipswich, MA, United States). The cDNA libraries were generated using the NEBNext Ultra RNA Library Prep Kit for Illumina (New England BioLabs) with NEBNext Multiplex Oligos for Illumina (New England BioLabs). All procedures were performed according to the manufacturer’s instructions. The quality and quantity of each library were analyzed using a High Sensitivity DNA Kit (Agilent) and subsequent quantitative PCR (KAPA), respectively. Sequencing was performed twice by the Genome Analyzer IIx (Illumina). The mRNA-seq data presented in this study were submitted to the DNA Data Bank of Japan Sequence Read Archive^1^ and can be retrieved via accession number PRJDB5797.

### Characterization of RNA-Sequencing Data

To characterize the transcriptome profile during RK formation, the obtained single read (SR) short reads (ca. 440 millions of 33-bp-length) were divided into each sample (ranges ca. 32–42 million reads) by a demultiplexing program supplied by Illumina and then mapped to the references described below using Bowtie (Langmead et al., 2009). The Cuffdiff2 (ver. 2.1.1) algorithm (Trapnell et al., 2013) was applied to obtain count data, to calculate the reads per kilobase per million mapped reads (RPKMs) and to identify Differentially Expressed Genes (DEGs). In this experiment only two biological replicates were performed, and the margin of error may be underestimated. In order to remedy this problem, we focused only on DEGs that show robust (twofold) changes for further analyses. The reference genome sequences (fasta) with annotation information (gff) for *A. thaliana* was downloaded from the FTP sites of Ensembl Plant^2^ [Release 22].

The analysis of vascular-related genes was performed as follow. The obtained SR short reads were divided into each sample by a demultiplexing program supplied by Illumina and then mapped to the references described below using TopHat2 (ver. 2.0.13) (Kim et al., 2013) to calculate RPKMs for each gene using Cuffdiff2 (ver. 2.1.1) (Trapnell et al., 2013). Procambium-, Ph- and xylem-related genes were picked then calculated fold change relative to non-infected roots.

### Comparison of RNA-Seq Results with Microarray Data

To characterize the transcriptome profile of RK, we compared our RNA-Seq data with two sets of publicly available microarray data for VISUAL leaf disks (Kondo et al., 2015) and for boron-treated suspension cells (Kubo et al., 2005). The MAS5-normalized microarray data for the VISUAL leaf disks (GSE61941) and those for the boron-treated suspension cells (GSE5748) were downloaded from NCBI GEO (Barrett et al., 2013). After transforming the data to the base-2 logarithm with a pseudo count of 0.001, the values were then normalized by the quantile method for each experiment using the R software (R Core Team, 2016). Multiple probe values were averaged to obtain a single gene expression value. As a compatible normalization procedure with this microarray data normalization, the gene expression values of the RNA-Seq data in this study were obtained as follows: the short read sequences were first mapped to the *Arabidopsis* NCBI RefSeq sequences (Brown et al., 2015) (downloaded on July 31, 2015) using Bowtie (Langmead et al., 2009). The count data for each sample were then transformed to the base-2 logarithm with a pseudo count of 0.125 and were then normalized by the quantile method. The count values for multiple transcript variants were summed to obtain a single gene expression value.

After converting the absolute gene expression values into the relative expression values by subtracting the average expression level of each gene in each experiment, we performed a complete linkage clustering based on Pearson’s distance in the samples using all the 17148 genes that were commonly available in both the RNA-Seq data in this study and the two *A. thaliana* microarray data.

### Quantitative RT-PCR

Total RNA were purified using the RNeasy plant mini kit (QIAGEN) from 7 dpi galls or non-inoculated roots (NIs), and cDNA was prepared using the Super Script III first strand synthesis kit (Invitrogen, United States). Real-time polymerase chain reaction amplifications were conducted using Light-Cycler 480II (Roche, United States) with the SYBR^®^ Green I Nucleic Acid Gel Stain (Roche) according to the manufacturer’s instructions.

Real-time PCR analyses were performed with the following primers: TMO6-qRT-F (5′- AACCTGCCGCCGAGAA -3′) and TMO6-qRT-R (5′- TGCCGGAAAAACAAAC -3′) for *TMO6*; ATHB8-F (5′- GCAAGCACGAGCAGCGATTCCC -3′) and ATHB8-R (5′- CTTGACCCCTCAACATCAGCCTC -3′) for *ATHB8*; TDR-qRT-F (5′- TGGTGGAAGTTACTTTGAAGGAG -3′) and TDR-qRT-R (5′- TCAATCTCTGTAAACCACCGTAA -3′) for *TDR*; and WOX4-F (5′- CCGGTCCGACAAAATT -3′) and WOX4-R (5′- CTCAATCTGTTGAGCA -3′) for *WOX4*. In all experiments, PCR values were normalized against those of the *GAPDH* gene, which was amplified using the primers GAPDHqRT-PCR-F1 (5′- TTAGTCGCAACCTGAAGCCATC -3′) and GAPDHqRT-PCR-R1 (5′- TTCCACTGCTACTTGAC CTTCG -3′). All of the qRT-PCR analyses were performed as biological triplicates. Student’s *t*-tests were performed to assess significant difference in mRNA levels.

### GUS Staining

The RKN-infected galls and non-infected roots were fixed in 90% acetone for 24 h at -30°C and stained in the GUS buffer with 0.5mg/ml 5-bromo-4-chloro-3-indolyl-β-D-glucuronidase (X-Gluc; Wako) for 1 h. The reaction was stopped with Carnoy’s solution (90% (v/v) methanol, 10% (v/v) acetic acid), and the roots were mounted in a chloral hydrate solution (8 g chloral hydrate, 2 ml water and 1 ml glycerol), observed using an Axio Imager M1 microscope (Carl Zeiss Microscopy) and photographed using a DP71 Digital camera (Olympus). GUS assays with *M. javanica* and *H. schachtii* were performed according to Cabrera et al. (2014).

## Results

### Histological Analysis of Gall Formation

We have established an *in vitro* infection experiment system using surface-sterilized RKNs (Nishiyama et al., 2015) and observed the infection processes in *Arabidopsis* (**Figure 1A**). In order to examine the growth dynamics of galls using the *in vitro* assay, we characterized the formation of galls over time at the cellular level. Multinuclear GCs were observed in 3 dpi galls, which became larger in 5 and 7 dpi galls as shown in **Figure 1B**. Small cells containing dense cytosol known as NCs were already present in 3 dpi galls. The stages of gall formation are defined by the size of GCs. 3D reconstruction analyses showed that 3 dpi *M. javanica*-induced *Arabidopsis* galls already contain GCs although hypertrophy was less apparent (Cabrera et al., 2015), (**Figures 1A,B**). However, GCs began to expand rapidly at 5 dpi, and by 7 dpi all GCs have entered the growth phase. In this respect, 5 and 7 dpi galls can be distinguished from that of 3 dpi (**Figure 1B**). Thus, the gall development process in the *in vitro* infection system used in this study is largely consistent with the events previously reported in the literature.

Given that RKNs first infect the vascular bundle in *Arabidopsis* roots then induce GC formation and NC proliferation, we decided to analyze the identity of vascular cells that contribute to the gall formation.

### A Comprehensive Transcriptome Analysis of Galls at Very Early to Medium Stages

**FIGURE 2:**
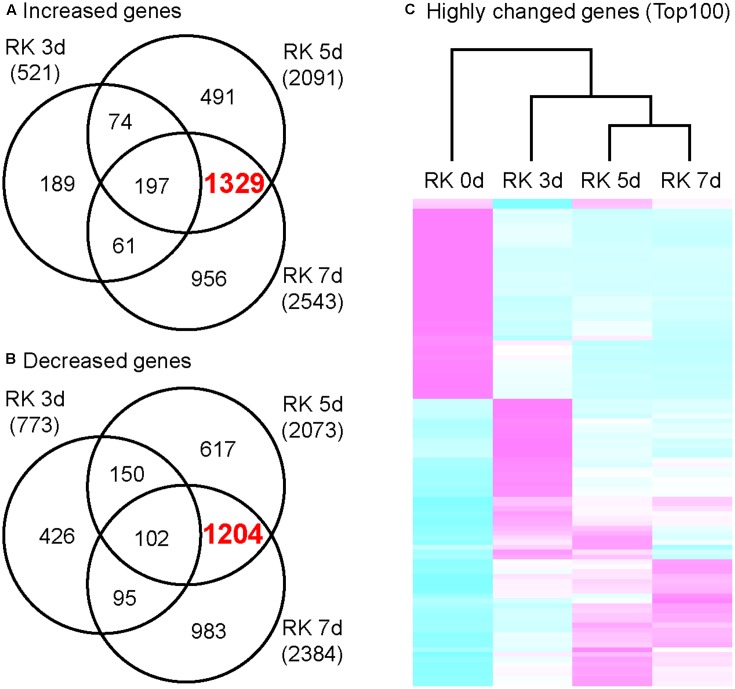
Transcriptome analysis of growing galls in the *in vitro* assay system. Venn diagrams that depict the number of genes increased **(A)** and decreased **(B)** in galls. Genes shown here all show at least twofold changes in expression relative to non-treated roots. **(C)** Comparison of differentially expressed genes that were determined by top 100 of FDR value. Magenta indicates genes increased compared to median levels of the four samples, whereas cyan means decreased amount of mRNA.

In order to characterize the global gene expression profiles in the gall during gall formation, we performed next-generation sequencing (NGS) analyses. Next, we calculated the amounts of mRNAs in galls, and selected 521 genes (3 dpi), 2091 genes (5 dpi), and 2543 genes (7 dpi) as highly up-regulated genes (greater than twofold increase relative to non-infected roots). Remarkably, the expression of 1329 genes were increased in 5 and 7 dpi galls but not in 3 dpi galls (**Figure 2A**). Similarly, 773 genes (3 dpi), 2073 genes (5 dpi), and 2384 genes (7 dpi) were selected due to their expression levels decreased to less than 0.5 fold relative to non-infected roots. Further, 1204 genes were down-regulated in 5 and 7 dpi galls but not in 3 dpi galls (**Figure 2B**). These results suggest that, the characteristics of transcriptome profile of 5 dpi galls generally resemble to that of 7 dpi galls, while the profile of 3 dpi galls is more distinct from either 5 or 7 dpi galls. Consistent with this, our clustering analysis with genes that were differentially expressed [Top 100 genes determined by False Discovery Rate (FDR)] at 7 dpi identified a cluster that includes 5 and 7 dpi profiles (**Figure 2C**). Our results suggest that the RKN infection and gall formation induce extensive transcriptional changes in 5 dpi galls. Given that gall formation was initiated before 3 dpi and cell proliferation was induced at 5 and 7 dpi (**Figure 1**), these transcriptome profiles may be associated with the newly produced cells in the galls at these time points.

### Procambium-Associated Genes Significantly Increased as Compared to Other Vasculature Genes

**FIGURE 3:**
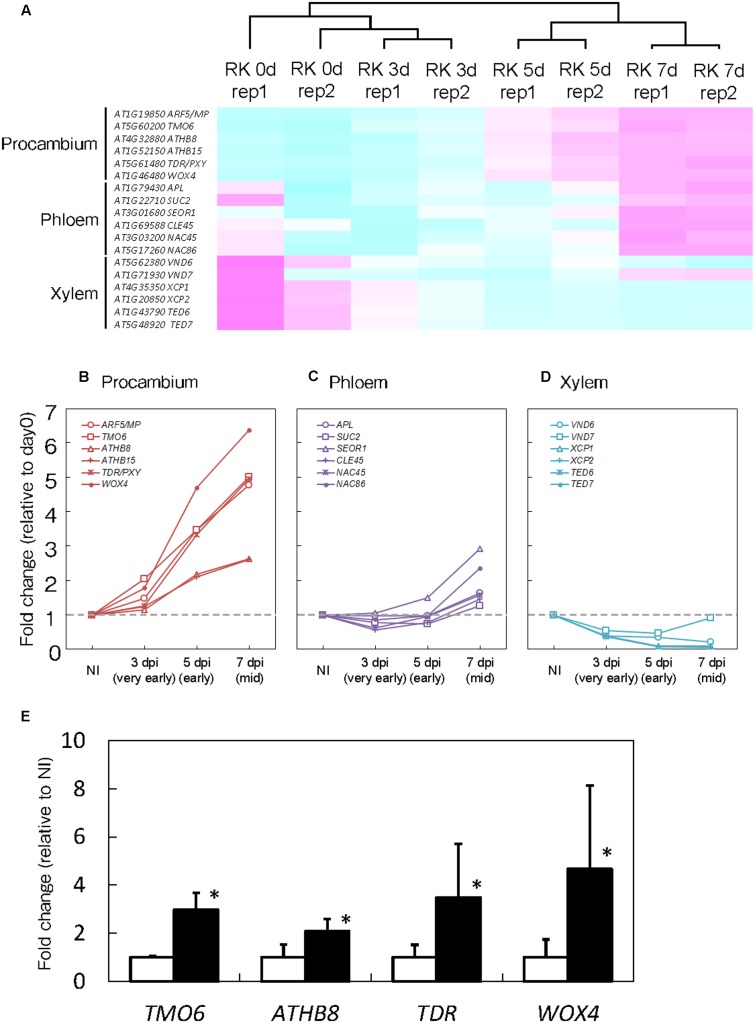
Procambium-associated genes are highly expressed in galls. **(A)** Comparison of genes that are associated with vascular cell identities. Magenta indicates genes increased compared to median levels of the four samples, and cyan means decreased amount of mRNA in the heat map. Time-course expression profiles of the procambium-associated **(B)**, Ph-associated **(C)** and xylem-associated **(D)** genes in the mRNA sequence analyses. The mRNA levels were calculated at each time point by normalizing to the mRNA level before infection. Values are the average of two experiments. **(E)** Quantitative RT-PCR results that denote the expression of *TMO6*, *ATHB8*, *TDR* and *WOX4* in 7 dpi galls and non-infected roots. Significant differences: ^∗^*P* < 0.05: values are means of three replicates ± SDs.

Root-knot nematodes first infect vascular cells in *Arabidopsis* roots where they induce the formation of GCs and proliferation of NCs (**Figure 1B**). Therefore, we decided to focus on transcriptional changes related to different vascular cell markers. Expression levels of *MP*, *TMO6*, *ATHB8*, *ATHB15*, *TDR* and *WOX4*, which are well-known procambium marker genes, significantly increased at 5 and 7 dpi (**Figures 3A,B**). In contrast, the mRNA levels of the Ph-associated genes such as *SUC2*, *APL*, *CLE45*, *NAC45*, *NAC86* and *SEOR1* (Truernit and Sauer, 1995; Bonke et al., 2003; Froelich et al., 2011; Depuydt et al., 2013; Furuta et al., 2014) showed only a small increment at late infection stages (**Figures 3A,C**). Transcript levels of xylem-associated genes such as *VND6*, *VND7*, *XCP1*, *XCP2*, *TED6* and *TED7* (Funk et al., 2002; Kubo et al., 2005; Endo et al., 2009) slightly decreased during gall formation (**Figures 3A,D**). Quantitative RT-PCR results further supported the notion that *M. incognita-*induced gall formation resulted in the accumulation of procambium-associated genes. The mRNA levels of *TMO6*, *ATHB8*, *TDR* and *WOX4* in the galls increased by 3.0-, 2.1-, 3.5-, and 4.7-fold compared with those of the non-infected roots, respectively (**Figure 3E**). Although transcriptome analyses of early GCs have shown similarities between GCs and vascular cells with *M. javanica*-induced galls (Barcala et al., 2010) and that *WOX4* is induced in of NCs in galls of *Medicago truncatula* (Damiani et al., 2012), the specific cell types in the vasculature that contribute to gall formation have not been determined. Our results suggest that the gene markers from the procambium, are rapidly activated at very early infection stages, suggesting a role for the procambium, but not the xylem or the Ph during gall formation.

### The Global Gene Expression Profile of Galls Is Similar to that of the Procambium

To demonstrate the close relationship between procambium formation and gall formation at the RNA level, we compared gene expression profiles of galls with previously published microarray analyses data from two xylogenic systems.

**FIGURE 4:**
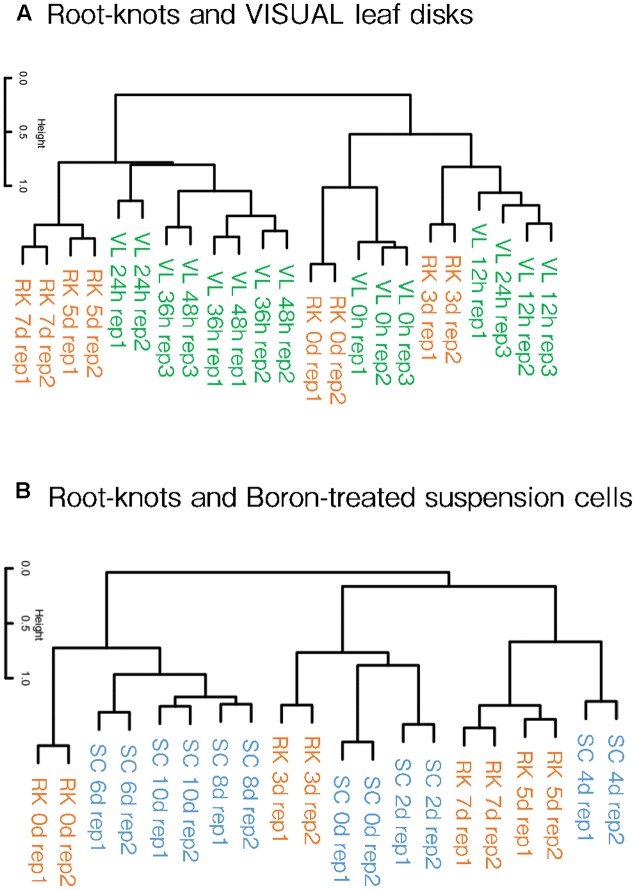
Hierarchical clustering of galls and artificial xylogenic system global expression profiles. Comparison of gall and VISUAL leaf disk transcriptome profiles published by Kondo et al. (2015), **(A)** and boron-treated xylem induction system published by Kubo et al. (2005) **(B)**. The complete data of the GEO accessions are shown in **Supplementary Table S1**.

Kondo et al. (2015, 2016) reported that differentiated cells can be converted into vascular cells through the VISUAL systems. The treatment of auxin, cytokinin and bikini following the application of TDIF converts mesophyll cells turn into procambial cells, which then assumes either procambium, xylem or Ph identities. Simultaneously, dynamic changes in the global gene expression profiles that accompany cell fate conversion were also described using this system. Our clustering analyses showed that the gene expression patterns of 5 and 7 dpi galls resembled those of 24, 36 and 48 h post-induction (hpi) VISUAL leaf disk expression profiles (**Figure 4A**). In contrast, 3 dpi gall expression profile formed a cluster with 12 hpi VISUAL leaf disk expression profile. In addition, the transcriptome profiles of the non-treated roots grouped together with untreated leaf disks. These results indicate that the clustering of gall maturation and long-term VISUAL treatment generated common characteristics.

Furthermore, we also compared our results with the microarray data of the boron-treated xylogenic systems that were published by Kubo et al. (2005). *Arabidopsis* suspension cells cultured with brassinolide and boron differentiated into xylem vessel elements. The gene expression profiles of 5 and 7 dpi galls formed a cluster with the expression profile of 4 dpi xylogenic system (**Figure 4B**). Importantly, procambium-related genes such as *TMO6*, *ATHB8* and *TDR* were transiently induced at 4 dpi but were reduced in later stages in the boron-treated xylogenic system (Kondo et al., 2015). In summary, gall cells are likely to share features similar to the procambium-like cells induced by the boron-treated xylogenic system. Moreover, the global expression pattern of 3 dpi galls forms a cluster with that of 0 and 2 dpi boron-treated suspension cells. This result suggests that galls in the very early stages of formation resemble undifferentiated suspension cells, whereas early-mid stage galls are more similar to the 4 dpi procambium-related cells. However, the transcriptome profiles of 6, 8, and 10 dpi xylogenic cells are not similar to those of the gall-forming cells at any stage.

### Procambium Cell Identity Markers Are Stimulated in Feeding Sites of Root-Knot and Cyst Nematodes

**FIGURE 5:**
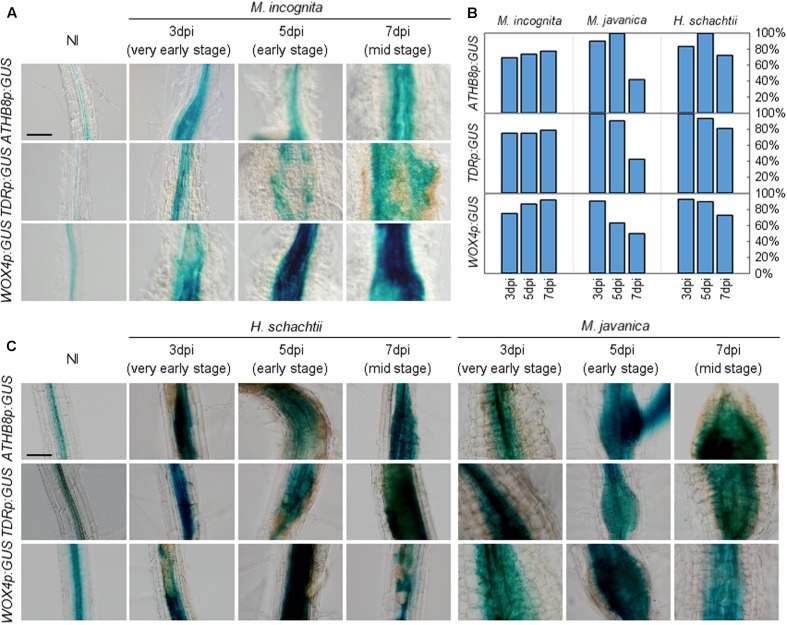
Promoter activities of procambium-associated genes are induced in galls. Five-day-old *Arabidopsis* seedlings harboring the *ATHB8p:GUS*, *TDRp:GUS*, or *WOX4p:GUS* constructs were inoculated with *M. incognita* **(A)**, *H. schachtii* or *M. javanica* **(C)**. GUS activity was detected in the vascular bundles of non-infected roots, while GUS signals were expanded in 3, 5, and 7 dpi galls within the vascular cylinder. Scale bar = 100 μm. **(B)** Penetrance of GUS staining in RKN-induced galls and *H. schachtii*-induced syncytia carrying the GUS reporter constructs, as some of the galls/syncytia did not show GUS staining. Bars denote the percentage of galls/syncytia that are GUS-positive (fully or partially stained with GUS).

To gain insights into the molecular basis underlying RKN infection, we explored whether RKN infections modify cell identities in root. The transgenic plants that carried the *ATHB8pro:GUS*, *TDRpro:GUS* or *WOX4pro:GUS* constructs displayed weak *ATHB8* and *TDR* promoter activities in the central cylinder of the *Arabidopsis* root (Baima et al., 1995; Hirakawa et al., 2010; **Figure 3A**). We found that the *ATHB8* and *TDR* promoters are more active during gall formation, as increased *ATHB8p:GUS* and *TDRp:GUS* signals were detectable in the 3 dpi galls, and the signals broadened considerably throughout the gall by 5 dpi (**Figure 5A**). Importantly, the GUS signals are restricted to the central regions of the root even though the root grows thicker over time, indicating that the *ATHB8*/*TDR*/*WOX4*-positive procambium-like cells increased in number in the vascular cylinder, and the proliferation of these cells may be responsible for the thickening of the roots (**Figure 5A**). However, GUS signals were not detected in all galls. Moreover, patchy GUS staining were occasionally observed, suggesting that not all gall cells express *ATHB8*, *TDR* and *WOX4* (**Figure 5B**).

We also assessed the relationships between phytoparasitic nematode infection and vasculature development by utilizing a different species of RKN *M. javanica*, and the CN *H. schachtii* to infect the *ATHB8*, *TDR*, and *WOX4* marker lines. Interestingly, the expression of these genes were also observed in both *M. javanica*-induced galls and *H. schachtii*-induced syncytia (**Figure 5C**). These results indicate that different species of endoparasitic nematodes (either RKNs or CNs) also activate the same procambium-associated genes in their feeding sites, which is similar to *M. incognita*. As we observed in the *M. incognita*-induced galls, GUS-negative cells and patchy GUS staining were also found in the *M. javanica*-induced galls and the *H. schachtii*-induced syncytia (**Figure 5B**). It is unclear why the staining in gall and syncytium were patchy, though it is possible that gene-silencing or protein degradation contribute to this pattern.

### Single Mutants of Procambium-Associated Genes Do Not Affect Galls Numbers

**FIGURE 6:**
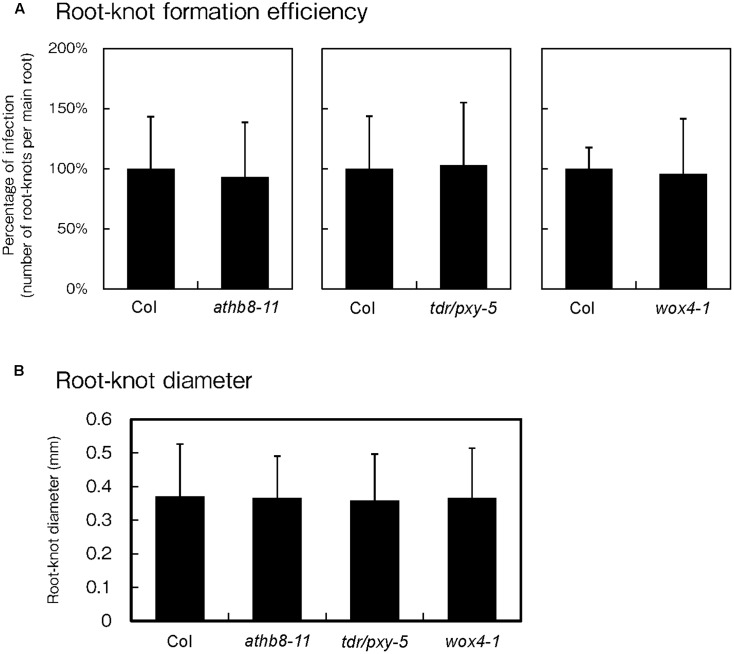
Effect of mutations on procambium-associated genes during *M. incognita* infection. Quantitative analysis of the gall numbers at 14 dpi **(A)** and gall diameters at 7 dpi **(B)**. Note that the comparison of gall numbers between the wild-type and the mutants was performed independently. The means of the percentages of infection from the mutant lines respect to the wild type controls are shown ± SDs.

Because phytoparasitic nematode infections activate procambial cell formation and induce the expression of procambium-associated genes in the feeding sites, the possibility that these genes regulate gall formation was investigated. To elucidate whether these genes are essential for gall formation, we examined infection efficiencies and gall growth using the *athb8*, *tdr* and *wox4* loss-of-function mutants. Based on our infection assay results, we found that these mutations did not cause visible effects during infection because the mutants produced equivalent number of galls to those of the wild-type plants (**Figure 6A**). We also measured galls diameters in these mutants and confirmed that the average gall sizes of the mutant lines were comparable to that of the wild-type (**Figure 6B**). These results suggest that single loss-of-function mutation in these genes are unlikely to affect gall formation processes. However, it is possible that single mutations alone may not be sufficient to completely abolish procambial cell formation. Furthermore, these genes have been reported to function redundantly (Hirakawa et al., 2008; Etchells et al., 2013), indicating that analyses with higher-order mutants will be necessary to determine whether these genes are required for procambial cell formation and gall formation.

## Discussion

### Vascular Stem Cells-Like Identity and Its Related Genes Are Induced during Gall Formation

Coordinated cell production and cell specification are fundamental processes in organogenesis of multicellular organisms. Small pools of stem cells are often present in restricted regions, and are responsible for cell proliferation that give rise to organs in the region. It is known that sophisticated molecular mechanisms are employed to regulate the number and activities of stem cells under normal growth condition. However, biotic or abiotic stimuli often affect these regulations (Yamaguchi et al., 2016).

Root-knot nematode-induced gall formation is an example of organogenesis triggered by exogenous biotic stimuli. Various studies have been performed to understand the characteristics of this plant-microorganism interaction. Recently, transcriptome profiling has been utilized as a powerful approach to identify genes and molecular mechanisms underlying nematode-induced organogenesis in *Arabidopsis* (Hammes et al., 2005; Jammes et al., 2005; Barcala et al., 2010). However, further analyses will be required to develop a more comprehensive picture of nematode parasitism and nematode-induced organogenesis.

Given that RKNs target vascular parenchyma cells to establish feeding sites (Bird, 1961; Bird and Koltai, 2000; Jones and Goto, 2011), we characterized the relationships between gall organogenesis and vasculature development using NGS-based transcriptomic profiling. Our clustering analyses with previously published xylogenic system microarray results suggest the gall global gene expression pattern closely resemble that of procambial cells. In particular, gene expression profiles of early- and mid-stage galls formed a cluster with that of 4 dpi boron-treated suspension cells (Kubo et al., 2005). Interestingly, even though boron-treated suspension cells express procambium-associated genes including *TMO6*, *ATHB8* and *TDR* at 4 dpi, their expression levels were reduced in later stages (Kondo et al., 2015). Accordingly, Barcala et al. (2010), reported that the global gene expression changes during GC formation in *M. javanica*-induced gall are similar to that of the boron-treated xylogenic systems (Barcala et al., 2010). In this study, we performed a more detailed analysis that includes different time points for both experiments. Hence, we identified the time points during galls formation that showed comparable transcriptomic profiles with particular stages of the boron-treated suspension cells, suggesting that gall cells display certain features similar to procambial cells. The expression profiles of early to mid-stage galls were distinct from that of 6 dpi suspension cells which closely resemble xylem, indicating that gall cells are dissimilar to cells with xylem identity.

The hypothesis that galls transcriptionally resemble procambium cells is also supported by the expression of several cell identity marker genes. Procambium-associated gene expression were clearly increased in galls while the expression of genes associated with xylem and Ph did not change significantly. In particular, expression of *TMO6* and *ATHB8* were indicative of procambium initiation during early stages of gall formation. Moreover, histological and histochemical analyses of lines expressing procambium-associated promoter:GUS constructs suggest that galls contain many RKN induced procambium-like cells. This unusual proliferation of small cells may account for the increase of the procambium-related gene expression in galls.

In addition to vasculature-related genes, *LBD16* is also expressed in the stele and lateral root primordia (Okushima et al., 2007), as well as GCs and proliferating NCs within galls during early infection, but the expression level decreases during mid-late stages (Cabrera et al., 2014). Moreover, loss-of-function *lbd16* mutant shows a reduction in the number of *M. javanica-*induced galls by ∼60% (Cabrera et al., 2014), suggesting LBD16 is required for successful gall formation. In the future, we should consider the importance of the relationship between procambium-associated genes and lateral root formation-related genes during RKN-induced gall formation.

### Molecular Basis of Procambial Stem Cell Induction and the Maintenance of Galls

Molecular machineries that regulate vasculature development may also be involved in gall formation. Vascular bundles are formed in the inner layer of the embryo along the direction of auxin gradient (De Rybel et al., 2016). The auxin-responsive transcription factor MP/ARF5 is known to be a central regulator of *Arabidopsis* embryogenesis that control various processes, including vasculature development initiation. MP regulates the expression of various downstream targets, including *TMO6* in the stele (Gardiner et al., 2010; Schlereth et al., 2010; De Rybel et al., 2016). In this study, we showed that genes related to vascular stem cell formation, including *MP*, are highly expressed in galls. This increased *MP* expression in galls is consistent with the transcriptome analyses for early GC performed by Barcala et al. (2010), and suggests molecular regulatory mechanisms analogous to procambium development may be employed in galls (Barcala et al., 2010). In this respect, GCs and NCs likely retain comparable cell identities during at least early stages of gall formation despite being morphologically distinct. Further, our promoter:GUS analyses of cells during *M. incognita* and *M. javanica* infection displayed comparable results, that procambium-associated genes are induced in galls formed by both species. The activation of procambium-associated genes is perhaps the potential common key process for GC and NC formation induced by RKN.

The TDIF-TDR signaling pathway and its downstream transcription factor WOX4 are known to be regulated by *MP* and play important roles in vascular stem cell homeostasis (Miyashima et al., 2013). Specifically, CLE family signaling peptide TDIF stimulates the expression of *WOX4* through its cognate receptor TDR (Hirakawa et al., 2010). These gene products are known to be positive regulators for the proliferation of procambial cells. Previous comparative genomic analyses suggested that phytoparasitic nematode genome contain genes that encode CLE-like peptides, and some of these CLE-like genes play critical roles during infection (Yamaguchi et al., 2016). Plant parasitic CN *H. glycine* and *Globodera rostochiensis* also secrete CLE-like peptides (Lu et al., 2009; Wang et al., 2010), and these peptides are predicted to modulate plant’s intracellular signaling pathway through LRR-type receptors for successful nematode infection (Guo et al., 2011; Replogle et al., 2011). Hence, the nematode CLE-like peptides are effector proteins secreted to the host cell cytoplasm mediate the parasitic process. Moreover, *M. incognita* has also been reported to produce CLE-like peptide 16D10, which is required for the infection processes (Huang et al., 2003, 2006a,b; Dinh et al., 2015). Although only phytoparasitic nematodes possess CLE-like peptides in the animal kingdom, the CLE peptide sequences diverge between RKNs and CNs. In addition, small signaling peptide phytosulfokines (PSKs) and the PSK receptor 1 (PSKR1) are also involved in the RKN infection (Rodiuc et al., 2016). Deciphering the contributions of these peptide hormone-related signaling pathways during parasitism requires further analyses.

Recently Guo et al. (2017), reported that CNs produce multiple types of CLE peptides and hypothesized that they utilize these peptides to hijack the TDIF-TDR-WOX4 signaling pathway. Consistent with these findings, our promoter:GUS analyses showed that CNs also induce *ATHB8* expression, which is known to act earlier to the TDR-WOX4 modules in vascular development, suggesting that the MP-dependent vascular developmental regulations were also activated in syncytium formation induced by CNs. Considering these observations, the possibility that CNs stimulate procambium initiation program similar to gall formation is likely. Understanding the molecular link between nematode effector proteins and the vascular development regulations will be the next critical task for this field.

Our findings suggest that phytoparasitic nematodes modulate plant’s developmental regulation of vascular stem cells to generate feeding sites during the infection. Subsequent works will hopefully identify the effector proteins responsible for these processes and lead to the answer of how nematodes modulate plant cells to produce galls and modulate GC and NC development.

## Author Contributions

YY NGS analysis. ReS GUS analysis, qRT-PCR. JC cyst nematodes. TS histological analysis. SN SE for NGS. ChE GUS analysis. RyS SE for NGS. YA SE for NGS. RO cyst nematodes. TK SE for NGS. TO SE for NGS. TD NGS analysis. TI, writing, SE for NGS, Arabidopsis, and RKN. CaE cyst nematodes. SS writing, Arabidopsis, and RKN.

## Conflict of Interest Statement

The authors declare that the research was conducted in the absence of any commercial or financial relationships that could be construed as a potential conflict of interest.

## Footnotes

1 http://trace.ddbj.nig.ac.jp/dra/index_e.html

2 http://plants.ensembl.org/index.html

## Supplementary Material

**TABLE S1 |** Microarray datasets used in this study.

## Abbreviations

CNs: cyst nematodes
GCs: giant cells
NCs: neighboring cells
PC: pro-cambial
Ph: phloem
PX: proto-xylem
RKNs: root-knot nematodes
RNA-Seq: RNA-sequencing
