# Supplementary material for: Root-Knot and Cyst Nematodes Activate Procambium-Associated Genes in Arabidopsis Roots
Source: Front Plant Sci. 2017 Jul 13;8:1195. doi: 10.3389/fpls.2017.01195 (PMC5506325; doi:10.3389/fpls.2017.01195)
Supplement: Supplementary file 1 [file Table_1.PDF]

Supplementary Table S1

Sample Name	Description	GEO accession
Root-knot formation of Col-0 roots.		
RK 0d rep1	Root-knots 0 day replicate 1	
RK 0d rep2	Root-knots 0 day replicate 2	
RK 3d rep1	Root-knots 3 days replicate 1	
RK 3d rep2	Root-knots 3 days replicate 2	
RK 5d rep1	Root-knots 5 days replicate 1	
RK 5d rep2	Root-knots 5 days replicate 2	
RK 7d rep1	Root-knots 7 days replicate 1	
RK 7d rep2	Root-knots 7 days replicate 2	
GSE61941 Time course microarray analysis during xylem cell differentiation in leaf-disk culture system.		
VL 0h rep1	VISUAL Leafdisk 0 hour replicate 1	GSM1517506
VL 0h rep2	VISUAL Leafdisk 0 hour replicate 2	GSM1517511
VL 0h rep3	VISUAL Leafdisk 0 hour replicate 3	GSM1517516
VL 12h rep1	VISUAL Leafdisk 12 hours replicate 1	GSM1517507
VL 12h rep2	VISUAL Leafdisk 12 hours replicate 2	GSM1517512
VL 12h rep3	VISUAL Leafdisk 12 hours replicate 3	GSM1517517
VL 24h rep1	VISUAL Leafdisk 24 hours replicate 1	GSM1517508
VL 24h rep2	VISUAL Leafdisk 24 hours replicate 2	GSM1517513
VL 24h rep3	VISUAL Leafdisk 24 hours replicate 3	GSM1517518
VL 36h rep1	VISUAL Leafdisk 36 hours replicate 1	GSM1517509
VL 36h rep2	VISUAL Leafdisk 36 hours replicate 2	GSM1517514
VL 36h rep3	VISUAL Leafdisk 36 hours replicate 3	GSM1517519
VL 48h rep1	VISUAL Leafdisk 48 hours replicate 1	GSM1517510
VL 48h rep2	VISUAL Leafdisk 48 hours replicate 2	GSM1517515
VL 48h rep3	VISUAL Leafdisk 48 hours replicate 3	GSM1517520
GSE5748 In vitro tracheary element transdifferentiation of Col-0 suspension cells.		
SC 0d rep1	Boron-treated suspension cells 0 day replicate1	GSM133956
SC 0d rep2	Boron-treated suspension cells 0 day replicate2	GSM133957
SC 2d rep1	Boron-treated suspension cells 2 days replicate1	GSM133958
SC 2d rep2	Boron-treated suspension cells 2 days replicate2	GSM133959
SC 4d rep1	Boron-treated suspension cells 4 days replicate1	GSM133960
SC 4d rep2	Boron-treated suspension cells 4 days replicate2	GSM133961
SC 6d rep1	Boron-treated suspension cells 6 days replicate1	GSM133962
SC 6d rep2	Boron-treated suspension cells 6 days replicate2	GSM133963
SC 8d rep1	Boron-treated suspension cells 8 days replicate1	GSM133964
SC 8d rep2	Boron-treated suspension cells 8 days replicate2	GSM133965
SC 10d rep1	Boron-treated suspension cells 10 days replicate1	GSM133966
SC 10d rep2	Boron-treated suspension cells 10 days replicate2	GSM133967
